# Multifunctional Graphene-Based Composite Sponge

**DOI:** 10.3390/s20020329

**Published:** 2020-01-07

**Authors:** Xu Cui, Jiayu Tian, Yin Yu, Aron Chand, Shuocheng Zhang, Qingshi Meng, Xiaodong Li, Shuo Wang

**Affiliations:** 1 College of Civil Aviation, Shenyang Aerospace University, Shenyang 110136, China; cuixu@sau.edu.cn; 2 College of Aerospace Engineering, Shenyang Aerospace University, Shenyang 110136, China; tianjiayu@email.sau.edu.cn (J.T.); yuyin@email.sau.edu.cn (Y.Y.); aaronchand714@yahoo.com (A.C.); zhangshuocheng@email.sau.edu.cn (S.Z.); mengqingshi@sau.edu.cn (Q.M.)

**Keywords:** graphene, sponge porous materials, multifunctional, flexible sensor, supercapacitor

## Abstract

Although graphene has been widely used as a nano-filler to enhance the conductivity of porous materials, it is still an unsatisfactory requirement to prepare graphene-based sponge porous materials by simple and low-cost methods to enhance their mechanical properties and make them have good sensing and capacitive properties. Graphene platelets (GnPs) were prepared by the thermal expansion method. Graphene-based sponge porous materials were prepared by a simple method. A flexible sensor was formed and supercapacitors were assembled. Compared with other graphene-based composites, the graphene-based composite sponge has good electrical response under bending and torsion loading. Under 180° bending and torsion loading, the maximum resistance change rate can reach 13.9% and 52.5%, respectively. The linearity under tension is 0.01. The mechanical properties and capacitance properties of the sponge nanocomposites were optimized when the filler fraction was 1.43 wt.%. The tensile strength was 0.236 MPa and capacitance was 21.4 F/g. In cycles, the capacitance retention rate is 94.45%. The experimental results show that the graphene-based sponge porous material can be used as a multifunctional flexible sensor and supercapacitor, and it is a promising and multifunctional porous nanocomposite material.

## 1. Introduction

In recent years, porous materials have been widely used in superhydrophobic materials, adsorption materials, electronics, and other fields owing to their porous, loose, and flexible characteristics [1,2,3]. Conductive porous materials have unique porous structure and demonstrate excellent electrical conductivity, enabling them to have a broad prospective application in various fields such as flexible sensing, electrochemistry, and supercapacitors [4,5,6]. However, conductive porous materials have their own limitations such as uneven pore size distribution and weak adhesion between the filler and pore structure. The porous materials prepared by pyrolysis [7], polymer solidification [8], or aerogel preparation [9] will produce low porosity and weak adhesion [10,11,12]. The preparation process of porous materials is complex, and their properties may be unstable. Compared with other porous materials, the pore distribution of sponge is uniform and its structure is stable [13].

In order to improve the conductivity and capacitance of porous sponge materials, conductive fillers are needed [14,15]. Among the commonly used carbon-based conductive fillers, graphene has been the most preferable nanofiller and has been extensively studied for its excellent electrical, mechanical [16,17,18], and capacitive properties [19,20,21]. Graphene has also exhibited excellent compatibility with polymers, making it one of the most promising candidates as nanofiller [22,23]. Graphene oxide and reduced graphene oxide can improve their dispersion in solvents by modifying the surface of graphene with functional groups, but their conductivity and structural stability are weaker than pristine graphene [24,25,26,27]. Compared with graphene, carbon nanotubes generally have the disadvantage of relatively weak ionic carrying capacity, which makes it difficult to improve the capacitive performance of polymers [28]. Therefore, graphene is an ideal conductive filler for porous materials. It is worth mentioning that graphene improves the capacitive properties of polymer very well. Graphene is a monoatomic layer of SP^2^ carbon atom with two-dimensional hexagonal crystal structure (HCC). Graphene is an ideal conductive filler for active materials of supercapacitors because of its strong ability to carry ions, hence it has attracted extensive research interest in the field of supercapacitors [29,30,31].

The combination of porous materials and fillers will greatly improve the mechanical properties, electrical conductivity and capacitance performance, which is worthy of further exploration [32,33,34]. However, there are still some unresolved problems. Some experiments have not studied the electrical responses of porous nanocomposites under tension, compression, bending, and torsion [35,36]. Yu Zhao et al. prepared flexible epoxy/Graphene platelets composite film using flexible curing agent J2000. The film has high tensile sensitivity, but the electric response under bending load is relatively weak, and the resistance change rate is 2% [37]. Yin Cheng, et al. developed a highly sensitive graphene-based fiber, which can be used to detect tensile strain, bending and torsion. The maximum resistance change rate of the graphene fiber can reach 140% when it is twisted in the anticlockwise direction, but the maximum resistance change rate is 40% when it is twisted in the clockwise direction [38]. The electrical response sensitivity of other types of graphene-based composite under bending and torsion loading may be weaker than that of graphene sponge. In situ polymerization of graphene sponge with manganese dioxide, poly (3,4-ethylenedioxythiophene), and carbon nanotubes can obtain high specific capacitance. However, the specific capacitance of pure graphene sponge obtained by relevant research is 9.78 F/g, so there is room for further improvement, and the study of capacitance properties of graphene sponge with different filler components was lacking [39,40,41]. In this experiment, these problems have been solved to a certain extent.

In this paper, a multifunctional flexible and conductive graphene-based sponge composite material is introduced. GnPs were prepared by thermal-sonication approach. The effects of different filler fractions on the mechanical and capacitive properties of nanocomposites were studied. The electrical responses of nanocomposites under different loading conditions such as tension, compression, bending, and torsion were tested. The electrical response of graphene sponge was measured when the temperature changed. GnPs synthesized by thermal expansion method are cheap and have good conductivity. The prepared sponge has good strength, sensing performance, and capacitance parameters. Graphene-based sponge composites have broad application prospects in multi-functional sensors and supercapacitors owing to their good versatility, easy production, and low cost.

## 2. Experimental Materials and Experimental Methods

### 2.1. Experimental Materials and Instruments

Asbury Carbons (Asbury, NJ, USA) kindly supplied graphite intercalation compound (GIC, 1721). The sponge made of melamine with the density of 15–17 kg/m3 was provided by Xijie Co (Wuhan, Hubei, China). The porosity was 88.725% ± 0.53%. Na2SO4 was purchased from Tianjin Hengxing Chemical Reagent Manufacturing Co., Ltd. (Tianjin, China). All chemicals were used as received without any further purification. 

Scanning electron microscope (SEM) images of the graphene sponge were obtained by SU8010 (Tokyo, Japan). Transmission electron microscope (TEM) images of the GnPs were obtained by JEOL JEM-2100 (Tokyo, Japan). Raman spectra of GnPs were characterized by Raman spectrometer Avantes AvaRaman (The Netherlands). Tensile test measurement was performed using a uniaxial tensile loading machine LTD GX-SF001 (Shenzhen, Guangdong, China). Fluke 2638A Hydra Series III Data Acquisition System (Everett, WA, USA) constantly monitored the resistance of the graphene sponge under different conditions. AnKe GDJS-408L high and low temperature test equipment is responsible for providing temperature environment for the change of resistance of graphene sponge with temperature. Capacitive properties of graphene sponge were analyzed using an electrochemical workstation ChenHua CHI660E B19038 (Shanghai, China). 

### 2.2. Experimental Methods

**Figure 1:**
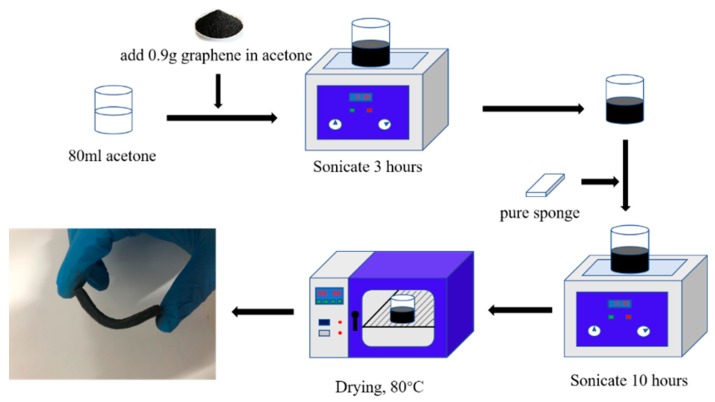
The fabrication of multifunctional graphene sponge nanocomposites.

A piece of sponge (rectangular cuboids of 6.0 cm × 2.0 cm × 0.3 cm) was used as the matrix of the composite material. Figure 1 shows the preparation method of 1.43 wt.% graphene sponge nanocomposites. GnPs were added to acetone to form a colloidal solution, which was then ultrasonicated for 3 h, resulting in the formation of a homogeneous GnPs solution. Afterwards, the sponge was soaked into the solution and simultaneously ultrasonicated for 10 h in order for the GnPs to permeate completely into the sponge skeleton. The composite sponge was dried in an oven at 80 °C for 5 h. 

Two sponge electrodes (6.0 cm × 2.0 cm × 0.3 cm) were immersed in 1 M Na_2_SO_4_ solution overnight. Then, a sandwich structure was created by combining the two electrode sponges, two pieces of platinum sheets as the current collectors, and a piece of filter paper as the separator.

## 3. Results and Discussion

### 3.1. Morphology of Graphene-Based Composite Sponges

**Figure 2:**
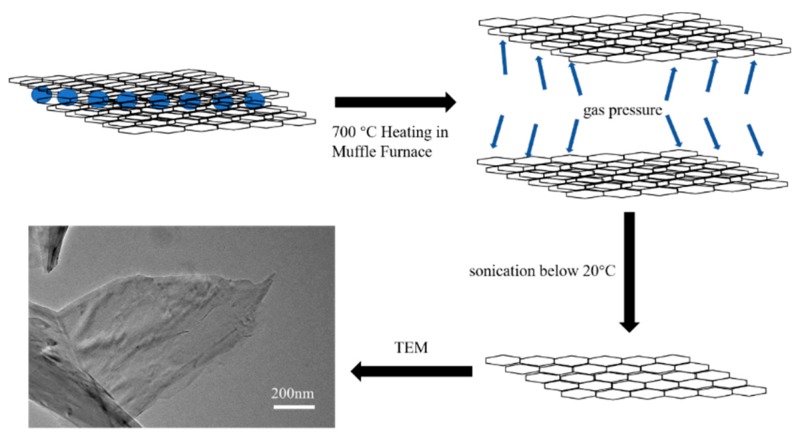
Schematic of thermal expansion process of graphite intercalation compounds. TEM, transmission electron microscope.

Heat treatment of graphite intercalation compounds (GICs) to produce thermal shock will immediately convert the intercalants into a large amount of gas, resulting in volume expansion. Figure 2 shows the expansion process from graphite intercalation compounds to the TEM image of graphene platelets. The graphite intercalation compounds were heat-treated in a muffle furnace at 700 °C for about 1 min. This rapid thermal expansion converts the dense, disk-like graphite GICs into worm-like expanded graphite flakes consisting of loosely jointed, thin GnPs [42]. Subsequent processing such as ultrasonication separates stacked graphene layers until a few layers remain, which is specifically known as GnPs. TEM image shows that the microstructures of GnPs have a large specific area and the platelets stack upon each other to promote electrical and thermal conduction. In addition, the platelet edges are featureless and very thin, thus possessing the characteristics of a monolayer graphene.

**Figure 3:**
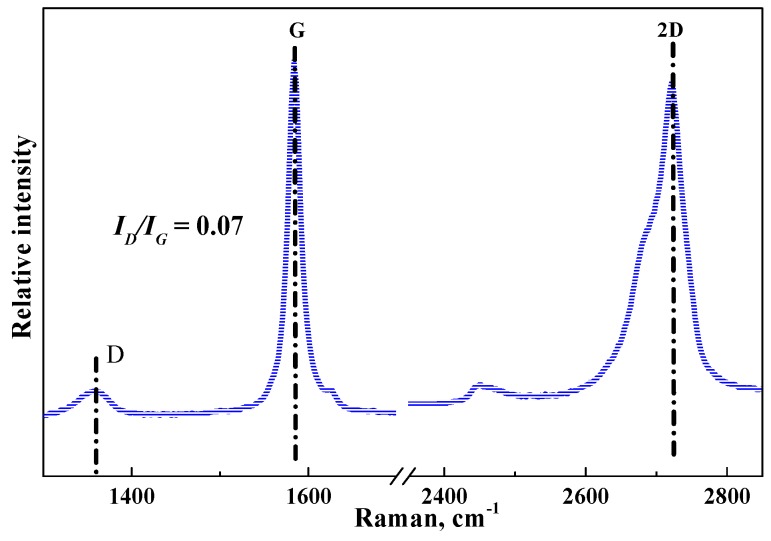
Raman Spectra of graphene platelets (GnPs).

In Figure 3, GnPs demonstrate obvious absorptions at 1359 cm^−1^, 1584 cm^−1^, and 2723 cm^−1^, which correspond to D, G, and 2D bands, respectively. G band refers to sp^2^ resonance on an ordered graphitic lattice, while D band is activated from the first order scattering process of sp^2^ carbons owing to the presence of in-plane defects such as substitutional hetero-atoms, vacancies, grain boundaries, or other defects, which might be sp^3^ hybridized carbon structure associated with the quantity of impurity or oxidation degree.

**Figure 4:**
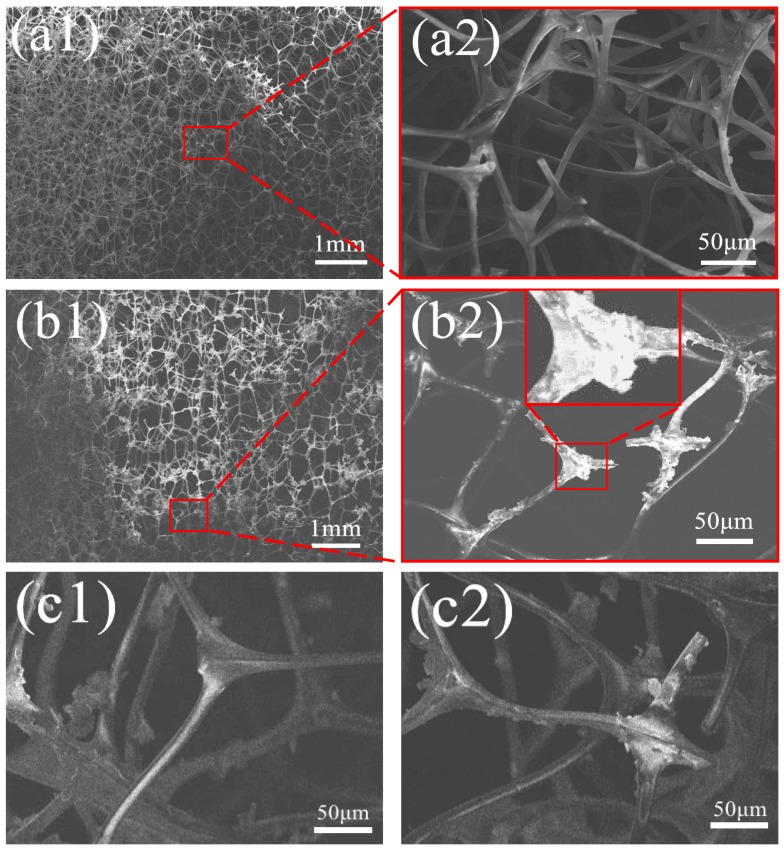
Scanning electron microscopic (SEM) images of the (**a1**,**a2**) pure sponge, (**b1**,**b2**) graphene sponge with ultrasonic vibration for 10 h, (**c1**) graphene sponge with ultrasonic vibration for 5 h, and (**c2**) graphene sponge with ultrasonic vibration for 15 h.

Figure 4 shows a comparison of scanning electron microscopic images of different sponges. The interconnected pores and the network structure of the sponge is clearly visible in Figure 4(a1,a2). Figure 4(a2) presents an extremely smooth surface which poses no barriers during GnPs’ deposition and the primary morphology of the sponge structure remains unchanged after coating. Figure 4(b1,b2,c1,c2) show the sponge morphology at filler fraction of 1.43 wt.%. The graphene sponges shown in Figure 4(b1,b2) were subjected to ultrasonic shock treatment for 10 h. Figure 4(b2) enlarges the representative areas of Figure 4(b1) at a high magnification. The SEM micrographs of the graphene sponge in Figure 4(b2) show a homogeneous dispersion of GnPs on the sponge pore surface, forming continuous electron pathways, which is essential for high performance electrodes. The quality of GnPs’ coating can be further improved by sonication during the dipping process. The graphene sponge shown in Figure 4(c1) was subjected to ultrasonic shock treatment for 5 h. The coating of graphene platelets on the sponge skeleton in Figure 4(b2) is more uniform than that in Figure 4(c1), and the amount of graphene platelets attached to the sponge skeleton is also greater. This is because the ultrasonic vibration time of the graphene sponge shown in Figure 4(c1) is shorter than that in Figure 4(b2). It is difficult to make more graphene platelets adhere to the sponge skeleton with shorter ultrasonic concussion time, and the uniformity of coating is poor. The graphene sponge shown in Figure 4(c2) was subjected to ultrasonic shock treatment for 15 h. In Figure 4(c2), the adhesion and coating uniformity of graphene platelets on the sponge skeleton are also not as good as that in Figure 4(b2). This is because the graphene sponge in Figure 4(c2) has undergone a longer ultrasonic vibration treatment than that in Figure 4(b2). Long time ultrasound may lead to the exfoliation of some graphene platelets that have been coated on the skeleton. Therefore, in this experiment, we choose 10 h as the ultrasonic treatment time of the graphene sponge.

### 3.2. Mechanical Property

**Figure 5:**
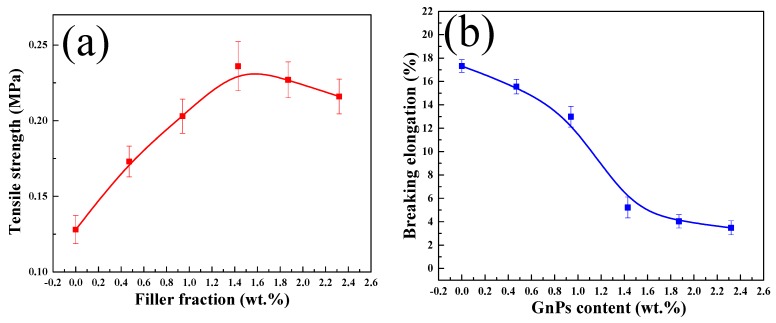
(**a**) The tensile strength under different filler fractions. (**b**) The breaking elongation with different filler fractions.

The tensile strength and elongation at break of sponges with different filler fractions are tested. Figure 5a demonstrates changes in tensile strength of sponge nanocomposites with the increase of GnPs filler fractions ranging from 0 wt.% to 2.32 wt.%. The maximum tensile strength of graphene sponge has reached 0.236 MPa at 1.43 wt.% filler fraction, depicting an increase by 45% as compared with that of the pure sponge. This increment is the result of the ability of the GnPs to provide more specific surface area and interfacial structure, which can effectively prevent stress concentrations and facilitate stress transfer across the interface under loading. However, the maximum tensile strength at 2.32 wt.% filler fraction is 8.47% lower than that at 1.43 wt.% of filler fraction. This may be because of the aggregation of graphene platelets with the increase of filler fraction, which leads to the decrease of the structural and mechanical properties of the attached sponge skeleton, thus reducing the maximum tensile strength of the graphene sponge.

As shown in Figure 5b, the elongation at break of sponge nanocomposites decreases with the increase of graphene filler fraction. When the graphene filler fraction is 2.32 wt.%, the elongation at break of the sponge nanocomposites decreases by about 79.9%. This is attributed to the enhancement effect of graphene on the sponge matrix, which experienced hardening and toughening effects during the accumulation of nano-fillers. In conclusion, GnPs increase the tensile strength and decrease the elongation of sponge nanocomposites.

By testing the mechanical properties, it was found that the sample with 1.43 wt.% filler fraction obtained the best tensile strength and preferable elongation. Therefore, 1.43 wt.% samples were selected as the representative of the follow-up series of experiments.

### 3.3. Tensile Linearity

Signal linearity is an important parameter to measure when determining the sensitivity of materials. The cross-sectional area of graphene sponge decreases during tension, but owing to the special high elastic skeleton structure of sponge, the distance between conductive paths decreases and the paths become denser in the process of cross-sectional shrinkage. These factors lead to a decrease in the resistance of graphene sponges during stretching. 

**Figure 6:**
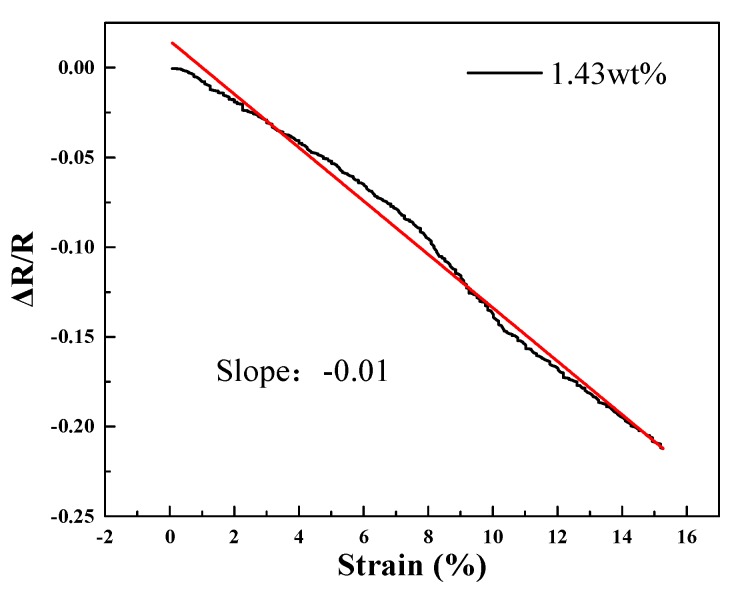
The stretch linearity at filler fraction of 1.43 wt.%.

As shown in Figure 6, the strain increases from 0% to 15.27% and the rate of change in relative resistance decreases uniformly with the increase of strain, which indicates that the material has a good linear response. The sensitivity coefficient (slope) is 0.01. On the basis of the aforementioned analysis, it can be highlighted that conductivity of the nanocomposite sponge is inversely related to the cross-sectional area of the specimen.

### 3.4. Electrical Response to Bending and Torsion

**Figure 7:**
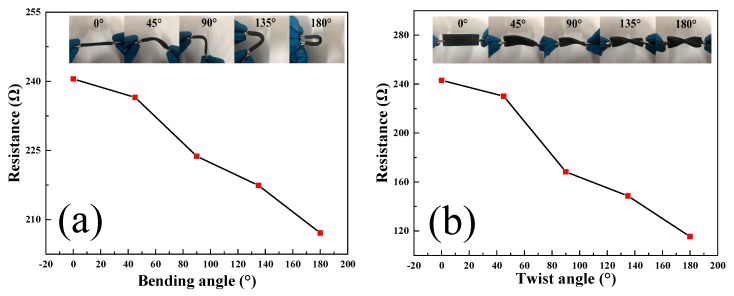
(**a**) The resistance variations with different bending angles; (**b**) The resistance variations with different twist angles.

The produced graphene-based sponge also possesses the ability of sensing deformations such as bending and twisting. Figure 7 shows the change in resistance at different bending angles and twist angles. From Figure 7, it can be seen that resistance changes at large angles are more sensitive than those at small angles, whether in torsion or bending. This may be because of the reduction in cross-sectional area, enabling the graphene sponge structure to become denser as the bending and twisting angle increases, which makes the sponge sensitive towards detecting angular deformations. Under 180° bending and torsion loading, the maximum resistance change rate can reach 13.9% and 52.5%, respectively. Whether the two loads are applied clockwise or counter clockwise, the electric response results are consistent. It can be seen that the graphene sponge has good electrical response performance under bending and torsion. This characteristic raises the possibility of application for this graphene-based sponge in the sensor and material health detection, especially when the sensorneeds to face bending and torsion and other complex loading methods.

### 3.5. Pressure Sensitivity

**Figure 8:**
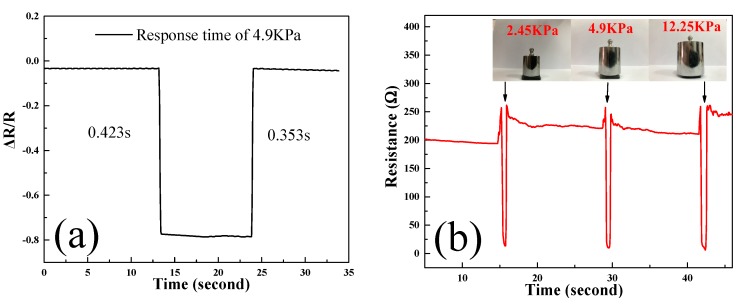
(**a**) Compression response time; (**b**) The pressure sensitivity testing of the GnPs sponge.

Figure 8a shows the reaction time of the composite material under 4.9 KPa. It can be seen that the composite material has a relatively short reaction time to pressure and has a certain pressure sensitivity. Further, the loads of 2.45 KPa, 4.9 KPa, and 12.25 KPa were applied to the graphene sponge, respectively, and the corresponding resistance changes were tested. As shown in Figure 8b, the resistance of the graphene sponge decreases under pressure. This is attributed to the compactness of the sponge’s skeleton and the denser GnPs, resulting in more conductive channels and lower resistance under compression. Resistance variation reaches a maximum at 12.25 KPa load. It is worth mentioning that the resistance value after unloading is close to the original resistance value, which reflects the good piezoelectric properties of graphene sponge and its application prospect as a pressure sensor.

### 3.6. Temperature Influence

**Figure 9:**
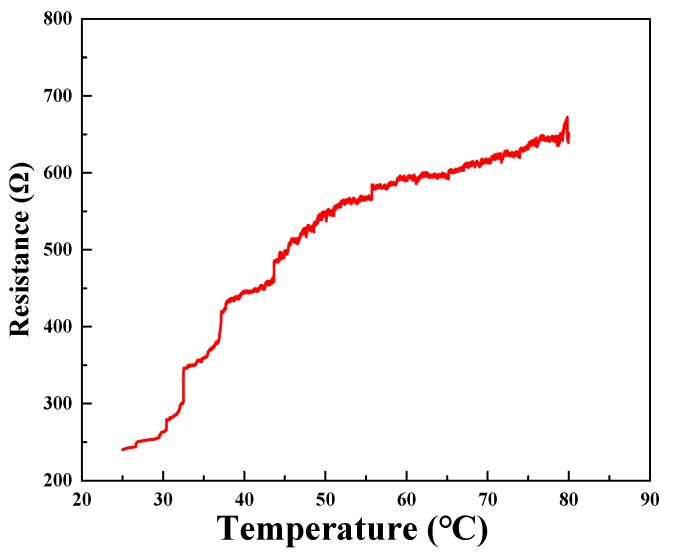
Change of resistance with temperature.

Temperature is one of the factors that affect the conductivity of nanocomposites. The change of ambient temperature will cause the change of semiconductor or conductor sensing parameters. Therefore, it is necessary to study the electrical response of graphene sponge under temperature change. In this experiment, the temperature range was 25 °C~80 °C, and the temperature rise rate of the composite was 50 s/°C. It can be seen from Figure 9 that the resistance changes greatly with the increase of temperature in the process of temperature rise below 50 °C. However, in the process of temperature rising above 50 °C, the change of resistance is relatively gentle. It can be seen that the sensitivity of the graphene sponge to temperature is relatively high in a specific temperature range, which may be because of the porous heat conduction structure formed by the graphene sponge. The electrical response of the graphene sponge at temperature needs further study.

### 3.7. Supercapacitor

The mechanical robustness and the distinctively porous structure of the graphene sponge imply that they have the potential to be used as electrodes for supercapacitor. The electrochemical performances of graphene sponge were studied using cyclic voltammetry (CV) and the galvanostatic charge/discharge (GCD) technique. All electrochemical measurements used two-electrode configuration, which can measure the cell performance more accurately.

**Figure 10:**
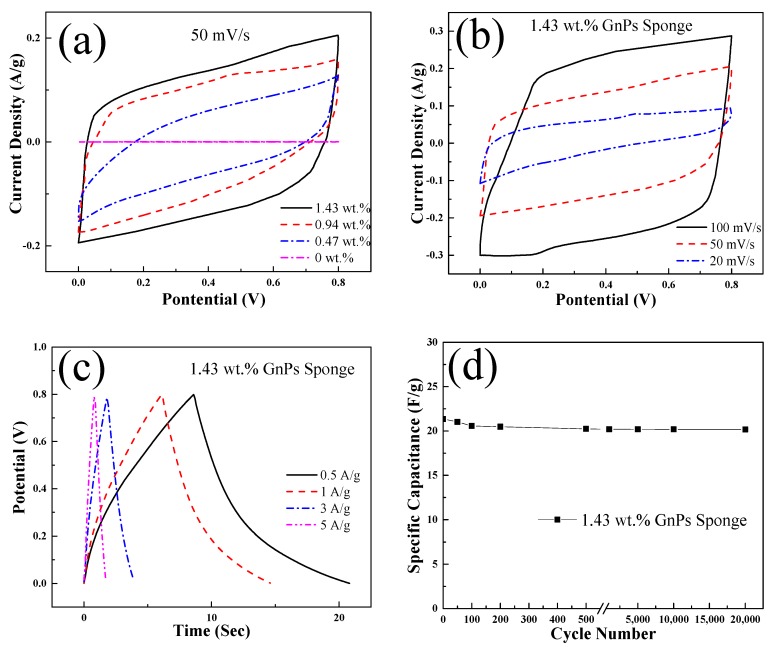
Electrochemical characterization of supercapacitors based on graphene sponge containing 1.43 wt.%, 0.94 wt.%, 0.47 wt.%, and 0 wt.% GnPs. (**a**) Cyclic voltammetry (CV) curves obtained at 50 mV/s. (**b**) CV curves at various scan rates. (**c**) Galvanostatic charge/discharge (GCD) curves of the 1.43 wt.% graphene sponge. (**d**) Cyclic stability.

Figure 10a shows the CV graphs of the graphene sponge electrodes at a scan rate of 50 mV/s within 0–0.8 V. For the pure sponge with GnPs filler fractions of 0 wt.%, CV curve is a straight line, implying no electrochemical capacitance. In comparison, the graphene sponge shows a near rectangular shape, implying a relatively high current response for double-layer capacitance behavior. This should be contributed by the GnPs that have feature high electrical conductivity. Therefore, the mechanisms of the supercapacitive performance can be classified as electrochemical double layer capacitances (EDLCs) [43].

The CV curve of the 1.43 wt.% graphene sponge is much closer to the rectangle-like shape than the curve of the other two, and thus the curve has a larger area, representing the highest specific capacitance. This is because of the fact that sponges with high GnPs fractions have more conducting channels and inter-particle pores.

EDLCs take important role in capacitance enhancement for the 1.43 wt.% graphene sponge. The specific capacitance values calculated from CV curves are 12.1, 13.8, and 16.7 F/g for the graphene sponge containing 0.47 wt.%, 0.94 wt.%, and 1.43 wt.% GnPs, respectively. The specific capacitance increases with GnPs filler fraction, because more GnPs create more electron pathways in the graphene sponge, leading to transfer of more electrons for electrochemical double layer capacitances.

The performance of the 1.43 wt.% graphene sponge was specifically investigated at 20–100 mV/s. In Figure 10b, the CV curves of graphene sponge electrodes maintain similar shapes at different scan rates, indicating remarkable electrochemical performance. The specific capacitances calculated from the CV curves are 12, 16.7, and 21.4 F/g when the scan rates increases from 20 mV/s to 100 mV/s, respectively. With the decrease of scanning speed, the specific capacitance of the graphene sponge increases slightly. This inter-testing rate capability is caused by the interconnected holes in the electrodes that affect the diffusion of electrolyte ions.

Figure 10c contains the GCD curves of the graphene sponge tested at 0.5, 1, 3, and 5 A/g. These graphene sponges show specific capacitance at 19.7, 16.5, 14.1, and 10.3 F/g of different current density from 0.5 to 5 A/g. It can be seen that the specific capacitance calculated from CV and GCD curves are similar. The GCD curves of 1.43 wt.% are almost linear and symmetrically mirrored to its discharge counterparts, which indicates a perfect electrochemical capacitive behavior. Similar to the specific capacitance obtained from the CV curve, which increases with the decrease in scanning rate, the specific capacitance obtained from the GCD curve also increases with the decrease in current density. The increments of capacitance with the decrease in either scan rates or current densities are caused by the time-dependent ion diffusions in the electrode materials [44].

The cyclic stability was measured for these graphene sponge electrodes. The capacitance decay was examined between 0 and 0.8 V at 0.5 A/g for 20,000 cycles. Figure 10d shows that capacity retentions are 94.45% for graphene sponge at 1.43 wt.%. Specific capacitance gradually decreases and remains relatively stable after the initial 2000 cycles. This can be attributed to porous structure evolved through the first few cycles to obtain a stable capacitance for the following cycles. The cyclic testing shows high stability for all the samples, which would be caused by the high structural integrity of graphene [45]. The fabricated graphene sponges have the potential to be used as electrodes for various energy storage devices.

## 4. Conclusions

In conclusion, GnPs were synthesized by thermal expansion and peeling. A multifunctional graphene-based sponge nanocomposite was prepared by a simple method. By studying and optimizing the composition of graphene filler, it was found that the mechanical properties and supercapacitance of sponge nanocomposites were the best when the filler composition was 1.43 wt.%. Under 180° bending and torsion loading, the maximum resistance change rate can reach 13.9% and 52.5%, respectively. The tensile linearity is 0.01488. The maximum tensile strength was 0.236 MPa and the maximum capacitance was 21.4 F/g. The sponge nanocomposites developed have good sensitivity and electrical response under bending, torsion, and compression loads. It shows good performance in flexible sensor and supercapacitor applications. The electrical response of graphene sponge at different temperatures was investigated. There are no expensive materials or complex equipment used in the whole manufacturing process of nanocomposites. It is believed that this material has broad application prospects in engineering, electronics, and other fields.
